# Investigating the Impact of Sorghum on Tau Protein Phosphorylation and Mitochondrial Dysfunction Modulation in Alzheimer’s Disease: An In Vitro Study

**DOI:** 10.3390/nu17030516

**Published:** 2025-01-30

**Authors:** Nasim Rezaee, Eugene Hone, Hamid Sohrabi, Rasheed Abdulraheem, Stuart K. Johnson, Stuart Gunzburg, Ralph N. Martins, W. M. A. D. Binosha Fernando

**Affiliations:** 1 Centre of Excellence for Alzheimer’s Disease Research & Care, School of Medical and Health Sciences, Edith Cowan University, Joondalup, WA 6027, Australia; n.rezaee@ecu.edu.au (N.R.); e.hone@ecu.edu.au (E.H.); hamid.sohrabi@murdoch.edu.au (H.S.); rasheeda@our.ecu.edu.au (R.A.); r.martins@ecu.edu.au (R.N.M.); 2 Centre for Ageing, Health Future Institute, Murdoch University, Murdoch, WA 6150, Australia; 3 Department of Biomedical Sciences, Macquarie University, Sydney, NSW 2109, Australia; 4 IngredientsbyDesign Pty Ltd., Lesmurdie, WA 6076, Australia; 5 CWEK Pty Ltd., South Fremantle, WA 6162, Australia

**Keywords:** Alzheimer’s disease, human tau, amyloid beta protein, mitochondria, sorghum, polyphenols

## Abstract

Background: Alzheimer’s disease (AD) is a progressive neurodegenerative disorder with poorly understood pathology. Elevated tau, phospho-tau and mitochondrial dysfunction are significantly correlated with an increased risk of AD and are therefore targets for disease-modifying therapy. In this study, we examined the effects of polyphenolic extracts from six different varieties of sorghum: Shawaya short black-1 (Black), IS1311C (Brown), QL33/QL36 (Red), B923296 (Red), QL12 (White), and QL33 (Red) on the attenuation of beta amyloid-induced phospho-tau levels, total tau levels, and mitochondrial dysfunction in neuronal cells. Method: Tau proteins (231 (pT231), Serine- 199 (pS199), and total tau proteins (T-tau)) were detected and quantified using sandwich ELISA kits, while mitochondrial dysfunction was measured in terms of mitochondrial membrane potential (Δψm) and adenosine triphosphate (ATP) levels. Results: Almost all varieties of the sorghum extracts reduced the beta amyloid-induced pS199 and pT231 levels (*p* ≤ 0.05). The optimum concentration of QL33/QL36 (1000 µg/mL), QL12 (2000 µg/mL), and QL33 (2000 µg/mL) strongly attenuated the phospho-tau level. Sorghum IS1311C (750 µg/mL) showed the highest Δψm reduction (39.8%), whereas QL33 (2000 µg/mL) most strongly improved the ATP level (37.7%) (*p* ≤ 0.01). For both Δψm and ATP assays, the least activity was observed in sorghum B923296 at 21% and 25.5%, respectively (*p* ≤ 0.01). Conclusions: The polyphenol extracts from sorghum attenuated the tau toxicity and Aβ-induced mitochondrial dysfunction in a variety- and dose-dependent manner and made a promising disease-modifying agent against AD. However, extensive research is needed to validate the efficacy of the sorghum extracts prior to animal and clinical studies.

## 1. Introduction

AD is characterised by the deposition of extracellular Aβ and intracellular tau proteins in the brain [1,2]. Accumulation of both proteins initiates a range of pathological events such as mitochondrial dysfunction, oxidative stress, loss of calcium regulation, and inflammation [3], leading to progressive loss of neurons [3]. Despite promising pre-clinical results, therapies which reduce plaque load have not been significantly helpful in clinical trials for AD [4,5].

Tau proteins are mainly present in neurons of the peripheral and central nervous systems [6]. Their main function is the stability of axonal microtubules, which is influenced by their phosphorylation state [6]. In AD brains, the function of the tau protein is disrupted, and the microtubule structures are damaged [7]. The phosphorylated tau dissociates from the microtubule and forms paired helical filaments (PHFs), which accumulate, resulting in the characteristic NFT seen in AD pathology [8]. Elevated phosphorylated tau (P-tau) levels at specific sites are more specific to AD pathology compared to total tau (T-tau), mainly reflecting the hyper-phosphorylated form in the brain tissue [9]. Serine199 (pS199)-tau and threonine 231 (pT231)-tau are two subtypes of phosphorylated tau proteins with different phosphorylated sites that are generated by different pathways [10,11]. Phosphorylation at Ser199 of Tau is considered to be an early event in the pathogenesis of AD [12]. Moreover, pS199 was suggested as a possible biomarker for the diagnosis of AD [13]. Other studies also suggest that threonine 181 (pT181), pT231, and pS199 are equivalent in their ability to discriminate AD from controls [14,15]. Furthermore, the importance of pT231 as blood biomarkers markers of AD has increased [16], where phosphorylation of tau protein at pT231 was shown to develop in the brain tissue of AD patients [17]. Moreover, it has been reported that elevated pT231-tau levels may also be a predictor of conversion from mild cognitive impairment to AD [18]. Thus, increased levels of pT231 may be a useful biological marker for the diagnosis of AD [19].

Mitochondrial dysfunction occurs at early stages of AD and evidence indicates that increased Aβ formation could impair mitochondrial function [3]. Examination of the AD post-mortem brain, as well as cellular and animal models, has demonstrated that Aβ initiates mitochondrial dysfunction via several pathways including elevation of ROS production, impairment of oxidative phosphorylation, interruption of mitochondrial dynamics, and interaction with mitochondrial proteins [20]. Amyloid precursor protein (APP) and amyloid Aβ accumulate in mitochondrial membranes prevent normal mitophagy, block mitochondrial protein transport, and degrade mitochondrial production, resulting in damage to mitochondrial structure and function [21,22]. Aβ may block the mitochondrial translocation of nuclear-encoded proteins [23] and components of the electron transport chain (ETC) [24,25]. Moreover, Aβ interacts with mitochondrial matrix components, such as enzymes of the Krebs cycle [26], Aβ-binding alcohol dehydrogenase (ABAD) [27], and presequence protease (PreP) [28]. The defect in the respiratory chain or the detoxification system can reduce electron transfer and ATP production and increase the accumulation of mitochondrial DNA (mtDNA) mutations and mitochondrial reactive oxygen species (ROS) production [29]. The unavoidable electron leakage during electron transfer causes the constant production of superoxide anions, which are responsible for 90% of the endogenous ROS [30]. Accumulation of ROS can lead to oxidative damage to nucleic acids, proteins, and lipids, another important hallmark of AD [29].

Naturally derived polyphenols (PPs) extracts have been reported to attenuate the overexpression of tau proteins and inhibit tau fibrillisation and aggregation through different pathways [31,32,33,34,35]. Polyphenolic extracts from black tea [36], blueberry [37], Ecklonia cava and Fenugreek [38] improve mitochondrial dysfunction by increasing mitochondrial membrane potential (Δψm) and decreasing mitochondrial ROS production [39]. Despite the high level of polyphenols in sorghum, its extract has not been previously investigated for attenuation of mitochondrial dysfunction.

In the present study, we investigated the effects of PP-rich extracts from six different varieties of sorghum against Aβ-induced T-tau/pT231/pS199 through the ELISA method in BE (2)-M17 cells. We further employed tetramethyl rhodamine ethyl ester (TMRE) and CellTiter-Glo assays to measure Δψm and ATP levels in the BE (2)-M17 cells.

## 2. Materials and Methods

### 2.1. Materials

Black clear bottom 96 well plate, Plate shaker, and Plate reader (PerkinElmer, EnSpire multimode plate reader, Waltham, MA, USA). The amyloid beta_42_ (Aβ_42_) was received from ERI Amyloid Laboratory, LLC (Oxford, CT, USA). The Micro BCA™ Protein Assay Kit, tau (Total, phospho (pS199), phospho (pT231)), Cell Lysis Buffer, Dulbecco’s modified Eagle medium/F-12 (DMEM/F-12) and GlutaMAX™ supplement, Hank’s balanced salt solution (HBSS), No phenol red (NPR) DMEM/F1, Fetal Bovine Serum (FBS), and Human ELISA Kit were purchased from Thermofisher Scientific™ Canberra, Australia. The Trypsin (sequencing grade), 1,1,1,3,3,3-Hexafluoro-2-propanol (HFIP) and dimethyl sulfoxide (DMSO) were obtained from Sigma Aldrich (Sydney, NSW, Australia). The TMRE mitochondrial membrane assay kit was obtained from (ABCAM, Melbourne, VIC, Australia). ATP standards were obtained from (Sigma Cat. #A7699). CellTiter-Glo was obtained from (Promega, G9241, Sydney, NSW, Australia).

### 2.2. Methods

#### 2.2.1. Sample Preparation and Polyphenol Extraction

Six different varieties of *Sorghum bicolor* (Shawaya short black-1 (Black pericarp), IS1311C (Brown pericarp), QL33/QL36 (Red pericarp), B923296 (Red pericarp), QL12 (White pericarp), and QL33 (Red pericarp) were obtained from Curtin University, Western Australia, Australia. Whole grain samples were ground for 5 min in a grain mill (CEMPTEC 1092, Foss Tecator, Höganäs, Sweden) until the flour passed 100% through a 500 µm sieve. The flour was stored in air- and moisture-proof packaging at −20 °C until the extraction process [40]. The polyphenolics were extracted in duplicate using 60% ethanol according to Svensson, Sekwati-Monang, Lutz, Schieber, and Gänzle (2010) [41] as optimised for sorghum [42]. Briefly, one gram of flour was mixed with 10 mL of 60% ethanol and then stirred in a water bath (Stuart Digital, DKSH, Melbourne, VIC, Australia) at 60 °C for 3 h. The mixture was centrifuged at 3220× *g* for 10 min at 4 °C and the supernatant was collected. The pellet was then resuspended in a fresh solution of 60% ethanol and centrifuged two more times, and all supernatants were pooled. The extracts were dried using SpeedVac (Thermo Scientific, SPD131DDA), after which the solid residue was resuspended in aliquoted 50 µL DMSO then stored at −80 °C until analysis.

#### 2.2.2. Preparation of Aβ Oligomer

The Aβ_42_ oligomers were prepared following a previous study [43]. Briefly, 1 mM Aβ_42_ peptide was prepared in HFIP, incubated for 30 min, aliquoted into 1 mL eppendorf^®^ tubes, and allowed to dry into film in a fume hood overnight. The peptide film was kept at −20 °C until required. Then, peptide films were redissolved in anhydrous DMSO and sonicated for 10 min. Thereafter, no phenol red F12 media was added to the final concentration of 100 μM, which was kept at 4 °C to incubate overnight before the treatment day [43].

#### 2.2.3. Cell Culture and Treatments

Human neuroblastoma BE (2)-M17 cells were cultured in DMEM/F12 supplemented with 10% (*v*/*v*) FBS and placed at 37 °C in a humidified incubator containing 5% CO_2_/95% air. Cells were allowed to attach for 24 h and then incubated to reach 80% to 90% confluency before the experiment. For treatment, cells were seeded on 12-well plates at a density of 10^5^ cells/well in growth media (DMEM/F12 supplemented with 10% (*v*/*v*) FBS). After 24 h, the growth media were removed and replaced by the associated treatment. Treatment groups included the positive control group (PC) (cells treated with 10 μM Aβ and treatment media (DMEM/F12 supplemented with 1% (*v*/*v*) FBS)), the negative control group (NC) (cells treated with treatment media without Aβ) and treatment groups (cells treated with sorghum extracts diluted in treatment media, 10 μM Aβ). Cells were incubated with treatments for 72 h. The Aβ concentration and test duration were chosen from the optimisation of the test conditions. The optimum concentrations of each sorghum extract (extracts diluted in treatment media) were used for this assay and determined based on the previously described dosage experiment [44]. These were determined to be 750 µg/mL, 750 µg/mL, 1000 µg/mL, 1000 µg/mL, 2000 µg/mL, and 2000 µg/mL for the Shawaya short black-1, IS1311C, QL33/QL36, B923296, QL12, and QL33 varieties, respectively. All concentrations are based on the initial weight of sorghum flour.

#### 2.2.4. Preparation of Cell Lysates

Cell lysate was prepared according to the manufacturer’s instructions. Briefly, the cell extraction buffer was mixed with a protease inhibitor cocktail (Sigma, P2714) (9:1) to make the lysis buffer. The cell media was removed and washed with HBSS containing calcium and magnesium. Then, 50 µL of lysis buffer was added to each well. The plates were kept on ice for 30 min, after which the cells were scraped, and the lysate was collected in microtubes. The supernatant was collected and transferred to another set of tubes after being vortexed for 10 min under 13 G and 4 °C.

#### 2.2.5. Measurements of Intracellular Tau and Phosphorylated Tau

After 72 h treatment and preparation of the cell lysate, the total protein levels were measured using a Micro BCA™ Protein Assay Kit according to the manufacturer’s protocol (Thermofisher Scientific, Australia). Briefly, samples were initially diluted by mixing 5 µL of cell lysate with 245 µL of PBS. The diluted samples, blanks, standards, and assay reagents were then added to a 96 well assay plate and incubated for 20 min in a 60 °C heating oven (Memmert, Schwabach, Germany). Then, the absorbance was read at 562 nm (PerkinElmer, EnSpire multimode plate reader, USA). The total protein was determined based on the standard curve.

T-tau and phospho-tau levels of treatment media were not detectable, which was expected, as the cell was kept alive during the treatment duration. Thus, evaluation of the tau level of cell lysate was the best option, as live cells do not release a considerable amount of tau in media.

Intracellular concentrations of total and phosphorylated tau proteins at pS199 and pT231 for the treatment and control groups were determined using the KHB0041, KHB7041, and KHB8051 ELISA kits from Thermo Fisher Scientific as per manufacturer’s protocols. Briefly, the ELISA standards were prepared, and cell lysates were diluted 10 times using a standard dilution buffer. For ELISA, samples and standards (100 μL per well) were added to the ELISA plate, covered, and incubated for 2 h at room temperature, then washed 4 times. The plate was then washed 4 times with the supplied wash buffer. Next, 100 μL of a detection antibody was added to each well and incubated for 1 h, then the plate was washed 4 times. The HRP solution was prepared and 100 μL was added to each well as they incubated for 30 min at room temperature and were then washed 4 more times. Next, 100 μL of chromogen was added to each well, and the plate was incubated for 15 min in the dark. Finally, 100 μL of stop solution was added to all wells, and the absorbance was read at 450 nm. Phospho- and T-tau values were determined using their respective standard curves. T-tau was read based on ρg/mL and P-tau was analysed based on Unit/mL. Tau levels were divided into total proteins to neutralise the possible effects of cell number and total protein in the interpretation of the results.

#### 2.2.6. Mitochondrial Membrane Potential (Δψm)

##### Optimisation of TMRE

TMRE assay measures the relative mitochondrial membrane potential in live cells through a fluorescence plate reader and fluorescence microscopy. The TMRE assay was optimised as follows: time points (4 h and 24 h), cell densities (1 × 10^4^ and 2 × 10^4^ cells/well), TMRE (0.5 µM and 1 µM), and Aβ (5 µM, 10 µM, 20 µM and 30 µM). The following sections briefly explain the assay protocol according to the manufacturer’s specifications for the 2.2.6.2 Δψm assay. The following conditions were selected for the TMRE assay based on the results of the optimisation and considering the higher difference in the control and treated group: 24 h, 20 mM Aβ, 1 µM TMRE, and 2 × 10^4^ cells/well. Exactly 24 h after plating, the cell culture medium (DMEM/F12 supplemented with 10% (*v*/*v*) FBS) was removed and replaced with the following treatments: The negative control group (NC) (cells treated with treatment media (DMEM/F12 supplemented with 1% (*v*/*v*) FBS) and the equivalent amount of DMSO and NPR f12 (without Aβ_42_, without extracts), positive control group (PC) (cells treated with 20μM Aβ_42_ without extracts), extract control group (EC) (cells treated with two doses of each extract, without Aβ_42_), extract-treated group (ET) (cells treated with two doses of each extract + 20 μM Aβ_42_), and blank group (treatment media or extract only). All the treatments including extracts and Aβ_42_ were added simultaneously. For this assay, one optimum dose and one lower dose (half of the optimum dose) were used: 750 µg/mL and 375 µg/mL for the extract Shawaya short black-1 and IS1311C, 1000 µg/mL, and 500 µg/mL for the extracts QL33/QL36 and B923296, and 2000 µg/mL and 1000 µg/mL for the extracts QL12 and QL33. Following the manufacturer’s instructions, before the end of treatment duration and staining with TMRE, FCCP was added to some negative control wells assigned to FCCP control treatment and incubated for 10 min. Thereafter, TMRE was added to the cells in the media and incubated for 20 min (10× TMRE was overlaid to the culture to reach the 1× concentration of 1 μM). After incubation, the media was aspirated and replaced with 100 μL of PBS/0.2% BSA for washing purposes (2 times). Then, fluorescence was on a plate reader with excitation-emission (Ex/Em) = 549/575.

##### Live Cell Imaging

Fluorescence imaging of live cells was performed on a parallel plate with the same treatment. Upon completion of the treatment duration and TMRE staining procedure, the medium was aspirated and replaced with 100 µL of PBS (twice). Images of mitochondrial membrane potential were taken by a fluorescence microscope (Nikon, Tokyo, Japan (Eclipse, Ti2-E)) in both brightfields and fluorescence modes.

#### 2.2.7. ATP Assay

##### Optimisation of CellTiter Glo ATP Detection Assay Kit

The ATP levels of the treated cells were measured using the CellTiter Glo ATP detection kit as per the manufacturer’s instructions (Promega). Adding cellTiter-Glo reagent to the cells results in cell lysis and generation of a luminescent signal, which can be interpreted as an indication of mitochondrial function and cell viability. The first step was the optimisation of the assay using three different time points (24 h, 48 h, and 72 h), Aβ (5 µM, 10 µM, 20 µM and 30 µM) and 1 × 10^4^ cells/well. This initial variable was chosen based on previous similar studies [44]. The assay was performed according to manufacturer instruction as explained in the following sections.

##### ATP Assay for Evaluation of Mitochondrial Function

After 24 h of plating, the cells culture medium (DMEM/F12 supplemented with 10% (*v*/*v*) FBS) was removed and replaced by the treatments groups: the negative control group (NC) (cells treated with treatment media (NPR DMEM/F12 supplemented with 1% (*v*/*v*) FBS) and the equivalent amount of DMSO and NPR f12 without Aβ_42_ and without extracts), positive control group (PC) (cells treated with 20 μM Aβ_42_ without extracts), extract control group (EC) (cells treated with two doses of each extract without Aβ_42_), extract-treated group (ET) (cells treated with two doses of each extract + 20 μM Aβ_42_), and the blank group (treatment media or extract only). Two different concentrations of each sorghum variety used were 750 µg/mL and 375 µg/mL for the extract Shawaya short black-1 and IS1311C, 1000 µg/mL and 500 µg/mL for the extracts QL33/QL36 and B923296, and 2000 µg/mL and 1000 µg/mL for the extracts QL12 and QL33. The plate and its content were equilibrated to room temperature for 30 min, and 100 μL of the thawed cellTiter-Glo reagent was added to each well of treated cells and mixed for 2 min on an orbital shaker to induce cell lysis. The plate was incubated at room temperature for 10 min to stabilise before the luminescent signal was read on a plate reader. A standard curve was generated from eight (8) different concentrations of ATP standards (4 µM, 2 µM, 1 µM, 0.5 µM, 0.1 µM, 0.01 µM, 0.001 µM, and 0.0001 µM), mixed with the cellTiter-Glo reagent for 2 min on an orbital shaker. After 10 min of incubation, the luminescent signal was recorded on a plate reader. Based on a formula derived from the ATP standard curve, each treatment’s ATP level was calculated after background readings were subtracted from cell-treated signals.

##### Statistical Analysis

Statistical analysis (ANOVA and Student’s *t*-test) was performed using SPSS version 22 (IBM, Armonk, NY, USA). Data were expressed as the mean ± standard deviation (SD) of three independent experiments. Comparison between treatments was performed using Tukey’s post-hoc test, where *p* ≤ 0.05 was considered statistically significant.

## 3. Results

### 3.1. The Effect of Polyphenol Extract from Sorghum on Aβ_42_-Induced Total-Tau

**Figure 1:**
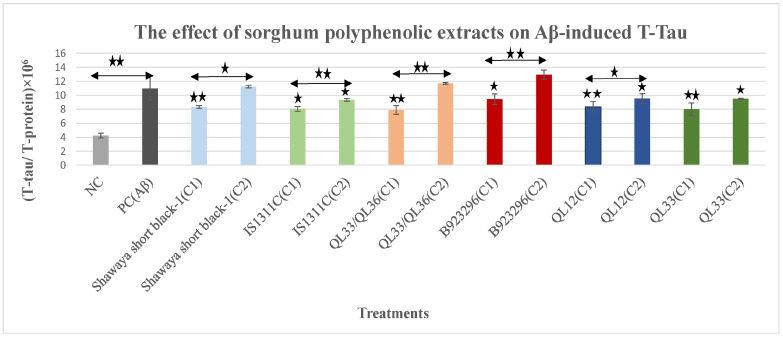
The effects of polyphenol extracts from six different varieties of sorghum in two doses on Aβ-induced T-tau levels of BE (2)-M17 cells. T-tau was normalised to the total protein level. All values were presented as the mean ± SD of three independent experiments (*n* = 3). Each experiment included two replicates. Statistically significant differences in comparison to the PC were marked (*) for *p* ≤ 0.05 and marked (**) for *p* ≤ 0.01. PC = positive control, NC = negative control, C1 = optimum concentration, C2 = half of optimum concentration (C1/2).

In the current study, the M17 cells were induced with Aβ_42_ to express tau as reported in previous studies [45,46,47]. Compared with the negative control cells, 10 μM Aβ_42_ treatment for 72 h significantly (*p* ≤ 0.001) increased the expression of normalised T-tau levels by 159% (Figure 1). Treatment with Aβ_42_ and sorghum extracts together reduced T-tau compared with the cells exposed to Aβ_42_ alone. Two concentrations (C1 and C2) of each sorghum extract were used to determine dose-dependent effects. We have previously reported that C1 is the optimum concentration [44] while C2 is a selected lower dose that is equivalent to half of each optimal dose (C_2_ = C_1_/2). The optimum doses of each extract are as follows (published previously): 750 µg/mL, 750 µg/mL, 1000 µg/mL, 1000 µg/mL, 2000 µg/mL, and 2000 µg/mL for Shawaya short black-1, IS1311C, QL33/QL36, B923296, QL12, and QL33. One optimum dose and one lower concentration (1/2 of optimum dose) were used for the Aβ/TBHP-induced ROS assays in order to assess the dose’s effects. At C1, significant reductions of Aβ-induced T-tau were observed in Shawaya short black-1 (*p* ≤ 0.01), IS1311C (*p* ≤ 0.05), QL33/QL36 (*p* ≤ 0.01), B923296 (*p* ≤ 0.05), QL12 (*p* ≤ 0.01), and QL33 (*p* ≤ 0.01) (Figure 1). The percentage of reduction of normalised T-tau was 24.2%, 26.6%, 28%, 13.9%, 24%, and 27%, respectively. For all the sorghum varieties, the C_1_ had a greater effect on T-tau reduction than C_2_. In fact, for B923296, the C_2_ did not show any significant effects (Figure 1). Among these six varieties, QL33/QL36 and QL33 showed the highest, while B923296 had the lowest reduction in induced T-tau levels (Figure 1). 

Figure 1 shows the effects of polyphenol extracts from six different varieties of sorghum in two doses on Aβ-induced T-tau levels of the BE (2)-M17 cells. T-tau is normalised to the total protein level. All values were presented as the mean ± SD of three independent experiments (*n* = 3). Each experiment included two replicates. Statistically significant differences in comparison to the PC were marked (*) for *p* ≤ 0.05 and marked (**) for *p* ≤ 0.01. PC = positive control, NC = negative control, C1 = optimum concentration, C2 = half of optimum concentration (C1/2).

### 3.2. The Effect of Polyphenol-Rich Extract from Sorghum on Aβ-Induced Phospho-Tau pS199

**Figure 2:**
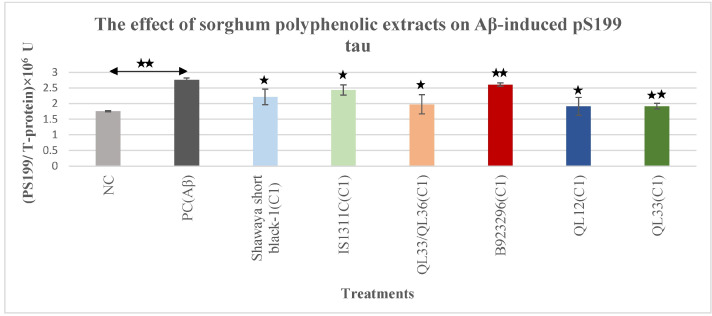
The effects of polyphenol extracts from six different varieties of sorghum on the Aβ-induced pS199 tau in BE (2)-M17 cells. The pS199 level was normalised to the total protein level. All values were presented as the mean± SD of three independent experiments (*n* = 3). Each experiment included two replicates. Statistically significant differences in comparison to the PC were marked (*) for *p* ≤ 0.05 and marked (**) for *p* ≤ 0.01. The presence of U in the graph is due to the relative nature of phospho-taus (unlike total tau, which has an absolute pg/mL value). NC = negative control, C1 = optimum concentration, C2 = half of optimum concentration (C1/2).

In this experiment, the Aβ_42_ treatment was used to induce cells to potentially increase phospho-tau [48,49]. Compared with the negative control cells, the 10 μM Aβ42 treatment for 72 h significantly increased the pS199 tau level (57%) (*p* ≤ 0.01) (Figure 2). When treated with an optimum concentration of sorghum extract, cells exposed to Aβ42 demonstrated a significant (*p* ≤ 0.01) reduction of pS199 tau compared with the cells treated only with Aβ_42_. The observed reduction of pS199 tau level with Shawaya short black-1 (*p* ≤ 0.05), IS1311C (*p* ≤ 0.05), QL33/QL36 (*p* ≤ 0.05), B923296 (*p* ≤ 0.01), QL12 (*p* ≤ 0.05), and QL33 (*p* ≤ 0.01) was 19.8%, 11.9%, 28.5%, 5.7%, 31%, and 30.6%, respectively (Figure 2). Among the six varieties of sorghum used in this study, QL12 and QL33 showed the highest reduction effect while B923296 had the least effect (Figure 2).

### 3.3. Effect on Aβ-Induced Phospho-Tau pT231

**Figure 3:**
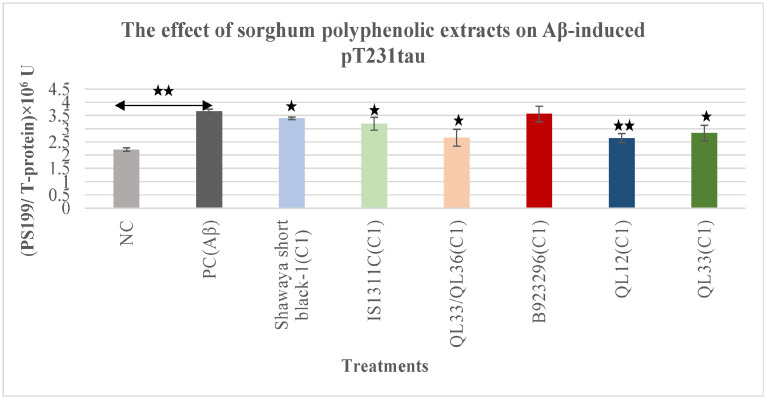
The effects of polyphenol extracts from six different varieties of sorghum on the Aβ-induced pT231 tau in BE (2)-M17 cells. The pT231 level was normalised to the total protein level. All values were presented as the mean ± SD of three independent experiments (*n* = 2). Each experiment included two replicates. Statistically significant differences in comparison to the PC were marked (*) for *p* ≤ 0.05 and marked (**) for *p* ≤ 0.01. The presence of U in the graph is due to the relative nature of phospho-taus (unlike total tau, which has an absolute pg/mL value). NC = negative control, PC = positive control, C1 = optimum concentration.

Compared with the negative control, 10 μM Aβ_42_ treatment for 72 h significantly increased the pT231 tau level (65%) (*p* ≤ 0.01) (Figure 3). The treatment of Aβ42 along with the optimum concentration of sorghum extracts significantly decreased pT231 phospho-tau level compared with the cells with Aβ_42_ treatment alone for QL12 (*p* ≤ 0.01), Shawaya short black-1 (*p* ≤ 0.05), IS1311C (*p* ≤ 0.05), QL33/QL36 (*p* ≤ 0.05) and QL33 (*p* ≤ 0.05) was 7.07%, 12.7%, 27.2%, 2.6%, 27.6%, 22. 3%, respectively. The QL33/QL36, QL12 and QL33 showed the strongest effect while B923296 had no significant reduction on pT231 level (Figure 3).

### 3.4. Effect of Sorghum Extracts on Mitochondrial Membrane Potential (Δψm)

**Figure 4:**
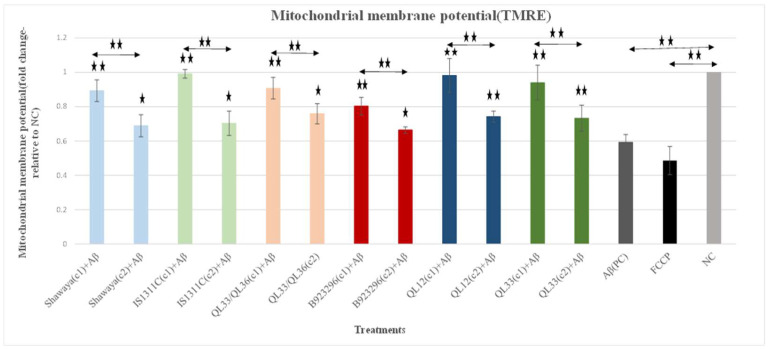
Attenuation effects of six different varieties of sorghum extracts on BE (2)-M17 cells against Aβ-induced Δψm loss. BE (2)-M17 cells were under treatment in different groups including control groups (PC and NC), assay control (FCCP), and extract-treated groups for different varieties/two different dosages for 24 h. The used concentrations of each variety were (750 µg/mL,375 µg/mL), (750 µg/mL, 375 µg/mL), (1000 µg/mL, 500 µg/mL), (1000 µg/mL, 500 µg/mL), (2000 µg/mL, 1000 µg/mL), and (2000 µg/mL, 1000 µg/mL) for Shawaya short black-1, IS1311C, QL33/QL36, B923296, QL12, and QL33, respectively. The Δψm was examined using the TMRE assay. Data are expressed as the mean ± SD (*n* = 3). Statistically significant differences in comparison to the PC were marked (*) for *p* ≤ 0.05 and marked (**) for *p* ≤ 0.01. Aβ exposure significantly reduces mitochondrial membrane potential.

TMRE emits a red fluorescence that can be detected by the spectrophotometric plate reader, flow cytometry or fluorescence microscopy in stained cells, and the level of TMRE fluorescence can be measured to determine whether mitochondria have high or low Δψm. Adding Aβ_42_ to the cells significantly decreased the Δψm level by 40.7% (*p* ≤ 0.01) (Figure 4). FCCP reagent has been used as a depolarisation control which causes low mitochondrial membrane potential and consequently lowers the TMRE signal. FCCP significantly reduced the Δψm signal by 51.4% (*p* ≤ 0.01) (Figure 4).

The PP-rich extracts were examined in two different non-toxic doses (higher and lower) [44] for their possible attenuation effects on Δψm loss. Extracts significantly attenuated the Δψm loss induced by Aβ_42_ (*p* ≤ 0.01) and (*p* ≤ 0.05) (Figure 4). Attenuation effects were stronger at higher doses (*p* ≤ 0.01) (Figure 4), indicating that the extracts exhibit dose-dependent attenuation effects.

**Figure 5:**
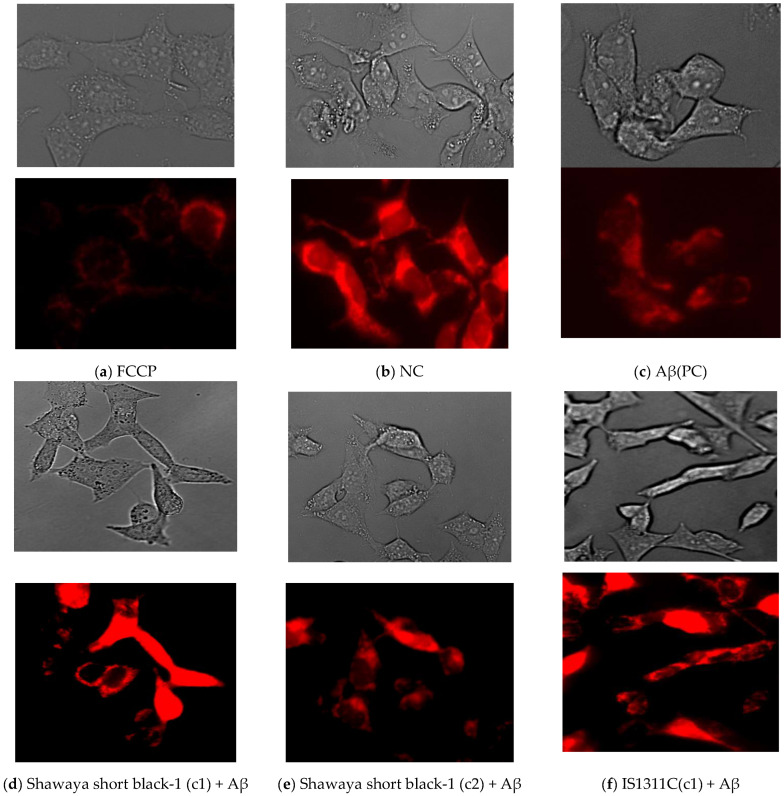

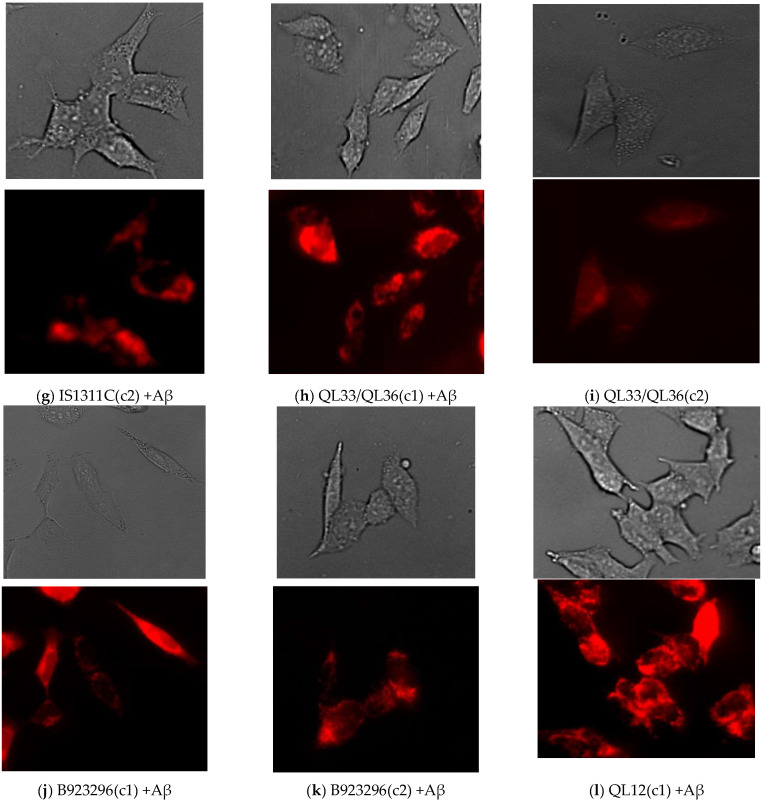

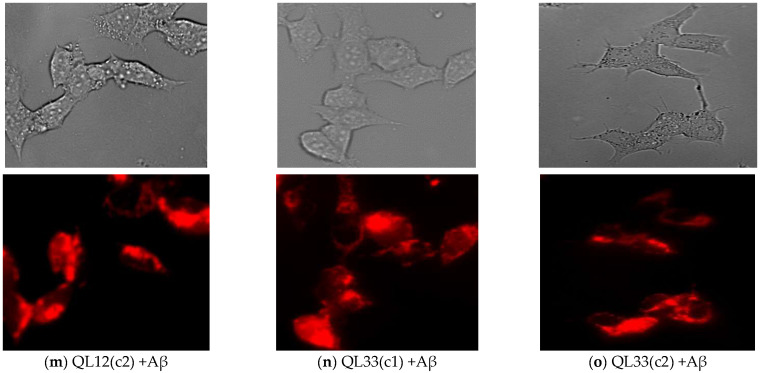
Bright field and fluorescence representative images of TMRE staining for each test group. (**a**) FCCP, (**b**) NC, (**c**) Aβ(PC), (**d**) Shawaya short black-1 (c1) + Aβ, (**e**) Shawaya short black-1 (c2) + Aβ, (**f**) IS1311C(c1) + Aβ, (**g**) IS1311C(c2) + Aβ, (**h**) QL33/QL36(c1) + Aβ, (**i**) QL33/QL36(c2), (**j**) B923296(c1) + Aβ, (**k**) B923296(c2) + Aβ, (**l**) QL12(c1) + Aβ, (**m**) QL12(c2) + Aβ, (**n**) QL33(c1) + Aβ and (**o**) QL33(c2) + Aβ. NC = negative control, PC = positive control.

Among the different varieties, sorghum IS1311C and B923296 showed the highest (39.8%) and lowest (21%) attenuation effects on Δψm loss (*p* ≤ 0.01) (Figure 5). The representative images of the TMRE-stained cells as a reflection of Δψm are shown in Figure 5. As expected, the fluorescence intensity of the images supports the results obtained from the plate reader. The red fluorescence intensity of TMRE decreased when treated with Aβ, which suggests the depolarisation of Δψm. Moreover, FCCP treatment also reduced the intensity of the red fluorescence compared to the untreated group (Figure 5). But this reduction was higher for the FCCP treatment group. Image observation also supports the quantitative results, suggesting that Aβ_42_-induced mitochondrial membrane depolarisation is significantly reversed by sorghum treatment in M17 cells, allowing for the integrity of the Δψm to be maintained (Figure 5). This effect is clearly visible when comparing Figure 5c,f.

### 3.5. Effect of Sorghum Extracts on ATP Level

**Figure 6:**
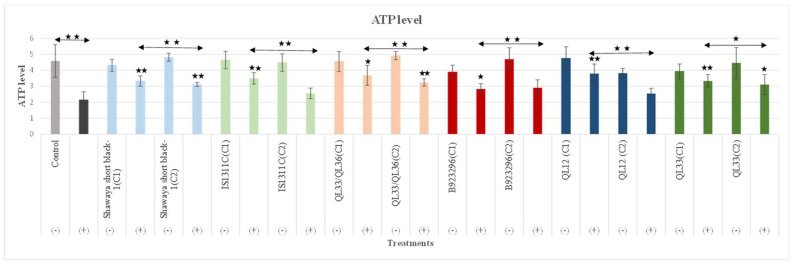
Attenuation effects of six different varieties of sorghum extracts in two different doses on BE (2)-M17 cells against Aβ-induced mitochondrial dysfunction (ATP loss). Cells were under treatment in different groups including control groups (PC and NC), extract control, and extract-treated groups for different varieties for 72 h. The used concentration of each variety was (750 µg/mL,375 µg/mL), (750 µg/mL,375 µg/mL), (1000 µg/mL, 500 µg/mL), (1000 µg/mL, 500 µg/mL), (2000 µg/mL, 1000 µg/mL), and (2000 µg/mL, 1000 µg/mL) for Shawaya short black-1, IS1311C, QL33/QL36, B923296, QL12, and QL33, respectively. The ATP examination was performed using the cellTiter-Glo assay. Data are expressed as the mean ± SD (*n* = 3). Statistically significant differences in comparison to the PC were marked (*) for *p* ≤ 0.05 and marked (**) for *p* ≤ 0.01. ATP levels decreased significantly after exposure to Aβ.

To further determine whether the PP-rich extract from sorghum is protective against Aβ_42_-induced mitochondrial dysfunction, we investigated the effects of these extracts on ATP levels using a Cell-Titer Glo assay kit, since Aβ-induced loss of Δψm could lead to the low efficiency of the proton motive force causing ATP depletion [50]. A significant reduction (52.8%) in total ATP was observed in the Aβ_42_ group (PC) compared to NC (Figure 6). The exact ATP (not relative) level was obtained using the standard curve of the ATP reagent. Two different doses (higher and lower) of each sorghum extract were examined for the ATP assay. The optimum concentration of all the varieties of extracts attenuated the ATP level (*p* ≤ 0.01) and (*p* ≤ 0.05). The percentage increases in ATP for the optimum concentration of each variety are 29.9%, 28.3%, 33.4%, 25.5%, 32.8%, and 37.7% for Shawaya short black-1, IS1311C, QL33/QL36, B923296, QL12, and QL33, respectively (Figure 6).

Among the six tested varieties of sorghum, QL33 and B923296 showed the highest (37.7%) and lowest (25.5%) effects on preserving ATP levels (*p* ≤ 0.01) in a dose-dependent manner (*p* ≤ 0.01) and (*p* ≤ 0.05) (Figure 6). The lower tested concentration of extracts was only significantly effective for Shawaya short black-1, QL33/QL36, and QL33 varieties (*p* ≤ 0.01) and (*p* ≤ 0.05) (Figure 6). Shawaya short black-1 (750 µg/mL) and IS1311C (750 µg/mL) are stronger than QL12 (1000 µg/mL) and QL33 (1000 µg/mL), likely due to their higher PPs content, which can be correlated with their darker colours, although they are less efficacious than QL12 (2000 µg/mL) and QL33 (2000 µg/mL).

In summary, all results indicate that PP-rich extracts from different varieties of sorghum inhibit Aβ_42_-induced taupathies and mitochondrial dysfunction in terms of Δψm and ATP levels as well as mitochondrial superoxide levels, which was shown in the previous study [44].

## 4. Discussion

The abnormal accumulation of hyperphosphorylated tau protein is a major contributor to neurodegeneration in AD and other tauopathies [51]. Tau is a microtubule-associated protein that relies on dynamic phosphorylation for its normal functions. In unhealthy conditions, it becomes hyperphosphorylated and toxic which could result in neurodegeneration [52]. In several neurodegenerative diseases such as Parkinson’s, frontotemporal dementia, dementia with Lewy bodies and AD, tau levels are highly elevated, which can be potentially used as a diagnosis and prognostic biomarker [53,54,55,56]. A high level of T-tau in cerebrospinal fluid (CSF) could be a sign of cortical atrophy in AD [57]. In a study by Pillai et al., 70 patients were evaluated within three years of the onset of AD symptoms. That study demonstrated the correlation between high levels of T-tau and early clinical signs of AD such as language or behavioural changes [57]. Tau phosphorylation at pS199 and pT231 has been implicated in AD neuropathology, which provides a possible target for the development of anti-tau agents that could potentially attenuate AD-related pathologies [13,16].

We have earlier reported that the PP-rich extracts of sorghum could decrease Aβ aggregation, Aβ-induced cellular death and oxidative stress [44]. As a sequel, the current study examined the effect of PP-rich extracts from six different varieties of sorghum on the level of T-tau, pS199 and pT231 through the ELISA technique. We showed that Aβ can increase the level of T-tau (159%), pS199 (57%), and pT231 (65%). This is consistent with previous studies indicating that Aβ induces T-tau and phosphorylated tau [49,58].

The sorghum PPs extracts decreased the level of Aβ-induced T-tau in a dose and variety-dependent manner (Figure 1). For both T-tau and phospho-tau assays, the optimum doses of extract were more effective than the lower doses, which also varied between the different sorghum varieties. For example, the reduction percentage of the T-tau level for the optimum dose of the sorghum Shawaya short black-1 was 24.2% while the lower dose did not show any reduction. In comparison, the reduction percentage of T-tau level for the optimum dose of the sorghum IS1311C was 26.6% compared to 14.7% for the lower dose. In addition, most of the tested sorghum varieties decreased the level of hyperphosphorylated tau at sites pS199 (5.7% to 31%) and pT231 (2.6% to 27.6%) (Figure 2 and Figure 3). The possible reason for greater effectiveness in sorghum variety QL33/QL36 and QL33 in T-tau reduction, QL12 and QL33 in pS199 reduction, and QL33/QL36 and QL12 in pT231 reduction could be the higher concentration of the optimum dosage for QL12 (2000 µg/mL) and QL33 (2000 µg/mL). Additionally, it could be due to the specific content of these sorghum varieties. For instance, sorghum QL33/QL36 has a relatively high level of Apigeninidin and Caffeic acid polyphenol [44], and sorghum extract QL12 possesses a relatively high level of Luteolinidin and caffeic acid, which has been shown to be effective in the attenuation of AD-related hallmarks such as oxidative stress, Aβ aggregation, mitochondrial dysfunction, and specifically, tau (for caffeic acid) [59,60,61,62,63]. Sorghum extract QL33 also has a high level of caffeic acid and ferulic acid, both of which have anti-tau activities through inhibition of tau phosphorylation and aggregation [59,64].

The other possible mechanism of action for the tau reduction effects of these sorghum extracts could be related to their effect on the attenuation of Aβ toxicity, inhibiting Aβ aggregation and increasing cellular viability [44]. It also may be due to the reduction of tau protein-paired helical filaments/fibrils through specific structural properties of PPs as well as their antioxidant activity through reducing cellular oxidative stress [65,66,67,68]. Moreover, sorghum PPs, specifically PPs with hydroxyl groups adjacent to aromatic rings, have the ability to prevent aggregation and disaggregate preformed Aβ fibrils, which could consequently affect the overexpression process of tau [67]. The mechanisms could be related to decreased GSK3β activity, an upstream signalling kinase of tau phosphorylation implicated in AD pathology [56,59,69]. Overall, sorghum extract has the potential to attenuate tau levels, specifically T-tau, pS199, and pT231, although more detailed molecular mechanism studies are required.

Furthermore, mitochondrial dysfunction is strongly implicated in the pathological process of AD [70,71]. However, the underlying mechanisms of mitochondrial dysfunction in AD are yet to be elucidated. In this study, for the first time, we investigated whether the polyphenol (PP)-rich extracts from sorghum could attenuate the Aβ-induced mitochondrial dysfunction. The speculation is that the PPs could restore the mitochondrial function affected by the administration of Aβ_42_ through different mechanisms, including manipulating mitochondrial dynamics and mitochondrial membrane potential, calcium load, the release of cytochrome c, endogenous enzymatic antioxidant capacity and restored oxidised protein levels, energy homeostasis, mitochondrial respiration complex I-IV, Nrf2/ARE/HO-1, and AKT/CREB/BDNF signalling pathways [72,73,74]. Mitochondrial ROS is a major contributor to mitochondrial function [75]. However, a high level of free radicals could damage the membrane phospholipids and DNA, leading to the loss of Δψm and apoptosis [76,77,78]. Moreover, there is a correlation between the ROS level and mitophagy at higher ROS levels [79].

In regard to the administration of Aβ decreased mitochondrial function in terms of Δψm and ATP (Figure 4 and Figure 6) (*p* < 0.01), the results align with the previous work [80], where the treatment of SY-SY5Y cells with Aβ significantly decreased Δψm compared to untreated cells (*p* < 0.05) [80]. Sorghum extracts significantly attenuated mitochondrial dysfunction in terms of increasing Δψm and ATP levels in a variety- and dose-dependent manner. This was consistent with our previous study [44], indicating the beneficial effects of these extracts on general and mitochondrial ROS levels. However, the sorghum B923296 (1000 µg/mL) did not significantly reduce ROS. It can be speculated that sorghum B923296 exerted its beneficial effects on mitochondrial function through pathways other than oxidative stress; this might also be associated with absence of Luteolinidin, which was present in all of the other five varieties tested. This PP is reported to have anti-inflammatory and antioxidant activities [63,81]. The IS1311C (750 µg/mL) and QL33 (2000 µg/mL) varieties were most potent in restoring the Δψm loss and ATP level (*p* ≤ 0.01) (Figure 4 and Figure 6). These effects are likely attributed to their ability to inhibit Aβ_42_-induced toxicity and oxidative stress. The high potency of sorghum QL33 in attenuating mitochondrial dysfunction was reported in our previous study [44]. This is probably attributed to its higher used concentration compared to other varieties: the optimum concentration of this variety was higher, and it possesses a polyphenolic profile that includes ferulic (55.79 ± 0.51 µg/g dry basis) and caffeic acids (34.68 ± 0.32 µg/g dry basis). Ferulic acid has improved mitochondrial biogenesis and dynamics markers in mice and human mononuclear cells [82], as well as decreasing the membrane fluidity of mitochondria and the ratio of cytochrome and upregulation of the expression of LC3-II, a marker of autophagy [83,84]. Caffeic acid, a potent PP present in all sorghum extracts, protects against mitochondrial dysfunction through different pathways, including decreasing free radicals, inhibiting mitochondrial permeability transition pores, and maintaining lipids and calcium [85,86,87]. Taken together, these data indicate that sorghum polyphenolic extracts significantly attenuate Aβ-induced mitochondrial dysfunction. As mitochondrial dysfunction has been known as a causative factor of synaptic failure in AD, its attenuation seems to be a promising avenue to protect synaptic strength and plasticity to consequently delay the cognitive decline process. However, future studies need to evaluate other mitochondrial functions and explore the molecular mechanisms behind these polyphenolic activities.

Despite these promising findings, this current study had a few limitations: crude polyphenol extracts contain substances like waxes, terpenes, and lipids, which could interfere with the polyphenols, and long-term toxicity and potential side effects of the polyphenol extracts were not assessed. Thus, the optimal doses reported cannot be directly translated to physiological doses in humans.

## 5. Conclusions

In this study, PP-rich extracts from sorghum demonstrated neuroprotective effects against Aβ-induced total/phospho tau in neuroblastoma M17 cells. The PP-rich extracts also showed strong neuroprotective effects on mitochondrial function (Δψm and ATP) in a variety- and dose-dependent manner. Nevertheless, these results warrant further comprehensive investigation in other cell and animal models to validate and confirm the neuroprotective effects of sorghum extracts.

## Data Availability

The data presented in this study are available on request from the corresponding author.
